# Book Reviews

**Published:** 1872-01-01

**Authors:** 


### Book Reviews.

A Text Book of Pathological Histology: An introduction to the study of Pathological Anatomy, by Edward Rindfleisch. Translated from the Second German Edition, with permission of the author, by William C. Kloman, M. D.; assisted by F. T. Miles, M. D., Prof. of Anat., Univ. of Maryland, with 208 illustrations. Published by Lindsay & Blakiston, Philadelphia. Chicago: For sale by Cobb, Andrews & Co. Price, $6.00.

This valuable and well-known work of Prof. Rindfleisch is now, for the first time, presented in an English translation. The rapid and important advances made during the past few years in the science of Pathology and Pathological Histology has been mainly owing to the researches of German investigators; and especially to the patient and careful labors of Profs. Rindfleisch and Billroth are we indebted for much of the knowledge gained in regard to these important subjects. The recent work of Prof. Billroth, translated by Hackley, being devoted exclusively to Surgical Pathology, touches but lightly upon the subject of Histology. Prof. Rindfleisch's work, therefore, fills as it were an unoccupied gap in our recent literature, and forms a most appropriate and valuable companion volume for the treatise of Prof. Billroth.

The Principles and Practice of Surgery. By John Ashurst, jr., M. D., Surgeon to the Episcopal Hospital, and to the Children's Hospital, Philadelphia. Illustrated, with 533 engravings. Henry C. Lea, 1871.

This is an octavo volume of 1011 pages. It takes up the subject in a systematic manner and treats it with as much thoroughness as the space permits. We observe one valuable addition, hitherto very much neglected by the authors of our surgical text books, and that is the appending to almost all the discussions of prominent operations a paragraph giving the statistical results hitherto observed. The work is well brought up to the latest improvements, and is worthy of the confidence of the profession.

An Introduction to Pathology and Morbid Anatomy. By T. Henry Green, M. D., Lond., Member of the Royal College of Physicians, etc. Illustrated by numerous engravings on wood. Philadelphia: Henry C. Lea, Publisher. For sale by Cobb, Andrews & Co., Chicago.

This is a small volume of 250 pages, intended mainly as an elementary text-book for the student. It embodies a brief account of the more important morbid processes which take place in the human body, in accordance with the present position of pathological knowledge, the views advanced being mainly derived from the works of the leading German investigators, Rindfleisch, Billroth, etc. The work while sufficiently comprehensive for the purposes for which it is intended, has the especial merit of presenting in a condensed form all the more recent advances in the science of Pathological Anatomy.

Cancer. Its Classification and Remedies. By J. W. Bright, M. D. Published by S. W. Butler, M. D. Philadelphia. Chicago: For sale by W. B. Keen & Cook. Price, $2.00.

Transactions of the American Opthalmological Society. Eighth Annual Meeting. July, 1871. New York: D. Appleton & Co.

The volume contains a number of valuable and interesting reports.

First Biennial Report of the Board of State Commissioners of Public Charities of the State of Illinois. December, 1871.

We acknowledge the receipt of the following volumes, which will receive notice in our next issue: Dalton's Treatise on Human Physiology. Pulmonary Consumption, by C. J. B. Williams, M. D., etc. Pulmonary Consumption, by James H. Bennet, M. D. Neuralgia, etc., by F. E. Anstie, M. D. Modern Therapeutics, Naphey's. The American Practitioner; edited by David W. Yandel, M. D.; vols. III and IV; 1871. The Physician's Pocket Record, comprising a Visiting List, many useful Memoranda, Tables, etc., by S. W. Butler, M. D., Philadelphia.

